# The BMP Pathway Participates in Human Naive CD4^+^ T Cell Activation and Homeostasis

**DOI:** 10.1371/journal.pone.0131453

**Published:** 2015-06-25

**Authors:** Víctor G. Martínez, Rosa Sacedón, Laura Hidalgo, Jaris Valencia, Lidia M. Fernández-Sevilla, Carmen Hernández-López, Angeles Vicente, Alberto Varas

**Affiliations:** Department of Cell Biology, Faculty of Medicine, Complutense University, Madrid, Spain; University of Iowa, UNITED STATES

## Abstract

Bone Morphogenetic Proteins (BMPs) form a group of secreted factors that belongs to the TGF-β superfamily. Among different roles in a number of immune cell types, BMPs are known to regulate T cell development within the thymus, although the role of BMP signaling in human mature T cells remains elusive. In this study, we demonstrate that canonical BMP signaling is necessary during two critical events that regulate the size and function of human naive CD4^+^ T cell population: activation and homeostasis. Upon stimulation via TCR, naive CD4^+^ T cells upregulate the expression of BMP ligands triggering canonical BMP signaling in CD25^+^ cells. Blockade of BMP signaling severely impairs CD4^+^ T cell proliferation after activation mainly through regulation of IL-2, since the addition of this cytokine recuperates normal T cell expansion after inhibition of BMP signaling. Similarly, activation of canonical BMP pathway is required for both the maintenance of cell survival and the homeostatic proliferation induced by IL-7, a key factor for T cell homeostasis. Moreover, upregulation of two critical receptors for T cell homeostasis, CXCR4 and CCR9, triggered by IL-7 is also abrogated in the absence of BMP signaling. Collectively, we describe important roles of the canonical BMP signaling in human naive CD4^+^ T cell activation and homeostasis that could be valuable for clinical application.

## Introduction

Bone Morphogenetic Proteins (BMPs) are multifunctional secreted growth factors that belong to the TGF-β superfamily together with TGF-β proteins, Activins and Inhibins, Nodal, Growth and Differentiation Factors (GDF), Miostatin and the anti-Mullerian hormone [1]. BMPs signal through heterotetrameric receptor complexes composed of two types of receptors. Among the type I receptors, ALK3/BMP receptor type IA (BMPRIA) and ALK6/BMPRIB are specific for BMPs, while ALK1/Activin receptor type IA (ActRIA) can bind both BMPs and Activins [2]. Similarly, the BMP receptor type II (BMPRII) only recognizes BMPs, while Activin receptor type IIA (ActRIIA) and IIB (ActRIIB) are able to recognize both BMPs and Activins [3]. The canonical BMP signaling pathway is initiated when the ligand-bound receptor complex phosphorylates the BMP receptor regulated Smad proteins (Smad-1, -5 and -8, termed BR-Smads as a group). Phosphorylated BR-Smads form a complex with the common Smad (Smad-4/Co-Smad) that is translocated to the nucleus where they regulate the transcription of several target genes. Alternatively, non-canonical signaling pathways can be triggered by BMP ligands depending on different factors such as the oligomerization of the heteromeric receptor complex [4].

First discovered by their capacity to induce ectopic bone formation [5], the BMPs are now known to play essential roles during embryonic development (reviewed in [6]) as well as in tissue homeostasis in the adult [7]. Regarding those organs that depend on BMPs for proper function, a considerable number of reports have established a pivotal role for BMPs regulating the differentiation of T cells within the thymic gland. In brief, BMP ligands are produced by both the thymic stroma and the CD34^+^ intrathymic precursor cells, which also express the components needed for BMP signaling. The BMP pathway blocks T cell differentiation at the CD4^-^CD8^-^ double negative to CD4^+^CD8^+^ double positive transition and maintains the intrathymic precursors by increasing their survival and inhibiting their proliferation [8–11]. The relationship between BMPs and T cells seems to continue during the mature stage of these cells, since a number of studies have described disparate responses induced by BMPs in differentiated T cells [12–14]. Most of these studies are based on mouse and cell line models, therefore the potential role of BMP signaling in human T cells has not been thoroughly addressed.

T cells constitute the main effector subset of the adaptive immunity. They are continuously generated in the thymus from where they emigrate to peripheral tissues as recent thymic emigrants [15]. When mature naive T cells confront their cognate antigen present on the surface of an antigen presenting cell, they become activated by signals transduced through the T cell receptor (TCR) and other costimulatory receptors such as CD28 [16]. Activation of T cells is characterized by a strong proliferative response accompanied by secretion of soluble factors.

During the steady state, contribution of the thymus to naive T cell repertoire maintenance is dominant at early stages of life but decays with age [17], whereas proliferation within the naive phenotype is dominant in older individuals [18]. Besides, naive T cells are characterized by a half-life of 414 ± 245 days depending on the technique employed [19]. According to these facts, it has been demonstrated that T cell homeostasis is regulated in the periphery by factors modulating their survival and clonal expansion such as IL-7 [20]. Furthermore, in different situations where lymphopenia takes place, such as neonatal thymectomy and HIV infection, IL-7 signaling is increased in order to reestablish basal T cell numbers [21–23]. Alterations in T cell number and function can lead to the generation of severe pathologies that range from immunodeficiencies, when T cell function is somehow inhibited, to chronic inflammation, when T cells show hyperreactivity.

In this study we show that activation of the BMP pathway is critical for human naive CD4^+^ T cell activation and homeostasis. Upon stimulation through the TCR, BMP production is induced in T cells acting in an autocrine fashion in the regulation of their proliferative response upstream to IL-2 signaling. Similarly, canonical BMP signaling is activated in response to IL-7 stimulation, mediating in the regulation of a number of features related to T cell homeostasis such as homing receptor expression, survival and homeostatic proliferation.

## Materials and Methods

### Cell culture

Buffy coats of healthy donors were obtained after written informed consent (Centro de Transfusión de la Comunidad de Madrid, Spain). The study was approved by the Ethics Committee for Clinical Investigation of the University Hospital Clínico San Carlos (Madrid, Spain). PBMCs were obtained by density gradient centrifugation with Lymphocyte Isolation Solution (Rafer, Madrid, Spain). Naive CD4^+^ T cells were isolated by negative selection using the Naive CD4^+^ T Cell Isolation Kit II (Miltenyi Biotech). Unless otherwise stated, 2-3x10^5^ T cells were cultured in 96-well flat-bottom culture plates in 200 μl of complete culture media, consisting in RPMI (Sigma-Spain) supplemented with 10% heat inactivated fetal calf serum (Invitrogen, Life Technologies), 1 mM pyruvate, 2 mM glutamine, 100 U/ml penicillin and 100 μg/ml streptomycin (all components from Sigma-Spain). For T cell activation, isolated T cells were cultured with immobilized anti-human CD3 (10 μg/ml) and soluble anti-human CD28 (4 μg/ml) monoclonal antibodies (ImmunoStep) or co-cultured with cytokine-matured monocyte-derived dendritic cells, generated as previously described [24]. Alternatively, isolated T cells were cultured in complete media alone or supplemented with PHA, a cytokine cocktail including rhIL-2 (25 ng/ml) (Miltenyi Biotech), rhIL-6 (100 ng/ml) and rhTNF-α (25 ng/ml) (Invitrogen, Life Technologies), or with rhIL-7 [5 ng/ml] (National Institute for Biological Standards and Control, NIBSC) alone. For determination of BMP2/4 and BMP6 expression by flow cytometry, brefeldin A (BioLegend) was added for the last 4 hours of culture. In some experiments, rhBMP2 or rhBMP4 (Humanzyme) was added to the cultures. Inhibition of BMP signaling was performed by addition of the recombinant human BMPR-IA/ALK-3 Fc chimera (R&D Systems) or the inhibitor molecule DMH1 (Tocris) at the indicated doses, while unspecific mouse immunoglobulins or vehicle (DMSO), respectively, were used as controls. The small inhibitory molecule DMH1 functions as an ATP-competitive inhibitor showing a very specific activity impeding Smad1/5/8 phosphorylation by ActRIA/ALK2 and BMPRIA/ALK3 in response to BMPs without affecting either non canonical pathways nor the TGF-β type I receptor/ALK5 activity [25, 26].

### PCR analysis

RNA isolation was performed using Absolutely RNA Microprep kit (Stratagene Cloning Systems), including a DNase I digestion step, as recommended by the supplier, to avoid genomic DNA contamination. Total cDNA was synthesized by High Capacity cDNA Reverse Transcription Kit (Applied Biosystems), according to the supplier’s instructions, and then used as target in the PCR amplifications. Real-time PCR was performed with the following Taq-man assays: *BMPR1A* (Hs01034909_g1), *BMPR1B* (Hs00176144_m1), *ACVR1A* (Hs00153836_m1), *BAMBI* (Hs00180818_m1) *SMAD1* (Hs00195432_m1), *SMAD4* (Hs00929647_m1), *SMAD5* (Hs00195437_m1), *SMAD8* (Hs00195441_m1) and *IL2* (Hs00174114_m1), all of them obtained from Applied Biosystems. *GNB2L1* (Pre-Developed TaqMan) was used as endogenous control. All PCR reactions were set in duplicates using the TaqMan Gene Expression Master Mix (Applied Biosystems) according to the manufacturer’s instructions. Amplifications, detections, and analyses were performed in a 7.900HT Fast Real-time PCR System (Centro de Genómica, Complutense University, Madrid, Spain). The Delta CT method was used for normalization to *GNB2L1* mRNA.

### Flow cytometry

The following mAb conjugated with FITC, PE, PE-Cy5 or APC were used for flow cytometric analysis: CD3 (HIT3a), CD4 (OKT4), CD25 (BC96), CD62L (DREG-56), CD49d (9F10), CD11a (HI111), CD18 (6.7), CXCR4 (12G5), CCR9 (BL/CCR9), CCR7 (3D12) and CD127 (R34.34) from BD Biosciences, Immunotech and BioLegend. Two-, three- and four-color immunofluorescence stainings were performed by incubating the cells in PBS containing 1% FCS and 0.1% NaN_3_ in the presence of saturating amounts of fluorochrome-conjugated antibodies for 30 min at 4°C. Staining with purified polyclonal anti-human BMPRIA (R&D Systems) was followed by incubation with fluorochrome-conjugated, multiadsorbed F(ab’)2 fragments of donkey anti-goat IgG (Jackson ImmunoResearch Laboratories).

For the intracellular stainings, and according to the manufacturer’s instructions, cells were treated with Cytofix/Cytoperm solution (BD Biosciences) for 20 min at 4°C, washed with Perm/Wash buffer (BD Biosciences), and stained with purified anti-human BMP2/4 mAb (100230), biotin-conjugated anti-human BMP6, purified anti-human BMPRIA (all from R&D Systems) or PE-conjugated anti-human Bcl-2 mAb (6C8) (BD Biosciences), followed when required by fluorochrome-conjugated, multiadsorbed F(ab’)2 fragments of donkey anti-goat, anti-rabbit or anti-mouse IgG or streptavidin (Jackson ImmunoResearch Laboratories), all diluted in Perm/Wash buffer. To detect phosphorylated BR-Smads, cells were fixed with BD cellFIX (BD Biosciences) for 30 min at 4°C to enable a phophorylation-state analysis. Then, cells were washed with PBS, permeabilized by 30 min incubation on ice with BD Phosflow Perm Buffer III (BD Biosciences) and stained with anti-human p-Smad1/5/8 polyclonal antibody (Ser 463/Ser 465) (Santa Cruz Biotechnology) followed by fluorochrome-conjugated, multiadsorbed F(ab’)2 fragments of donkey anti-rabbit IgG (Jackson ImmunoResearch Laboratories), all diluted in PBS. Analyses were conducted in a FACSCalibur flow cytometer (BD Biosciences) from the Centro de Citometría y Microscopía de Fluorescencia, Complutense University of Madrid.

### Immunofluorescence analysis

For immunofluorescence analysis, freshly isolated CD4^+^ T cells were attached to poly-L-lysine coated glass slides by 30 minutes of incubation at room temperature and stained with CD4 (RPA-T4) (BD Biosciences) and BMPRIA (R&D Systems) antibodies followed by Alexa Fluor 594 or Alexa Fluor 488-conjugated multiadsorbed F(ab’)2 fragments of donkey anti-mouse and donkey anti-goat IgG. For intracellular staining of BMPRIA, cells were fixed with Cytofix/Cytoperm solution (BD Biosciences) before CD4 staining and stained for BMPRIA using Perm/Wash buffer (BD Biosciences). Finally, cells were stained with Hoechst 33342 (Invitrogen, Life Technologies) for cell nucleus visualization and mounted with Prolong Gold (Invitrogen, Life Technologies). Slides were examined on a FluoView 1200 Confocal Microscope (Olympus) from the Centro de Citometría y Microscopía de Fluorescencia, Complutense University of Madrid.

### Proliferation assays

A specific kit from Roche Diagnostics, BrdU Labeling and Detection Kit III, was used to measure BrdU incorporation into newly synthesized DNA. Briefly, after 4 days of stimulation the cultures were pulsed for 12 h with 10 μM 5-bromo-2’-deoxyuridine (BrdU), the labeling medium was removed, and cells were dried (2 h at 60°C), fixed in ethanol in HCl (0.5 M) for 30 min at -20°C, treated with nucleases (30 min at 37°C), and then incubated with peroxidase-conjugated Fab fragments of mouse anti-BrdU (30 min at 37°C). The peroxidase reaction was developed with ABTS substrate, and the sample absorbance was measured using an ELISA reader (ELX800MB, Bio-Teck Instruments) at 405 nm with a reference wavelength at 492 nm. As an alternative, isolated naive CD4^+^ T cells were labeled with 5µM CFSE (Sigma-Spain) to determinate their proliferative response at the indicated time points by the CFSE dilution method. Determination by flow cytometry of the percentage of cells in each stage of the cell cycle was carried out using the fluorochrome Hoechst 33342 (Invitrogen, Life Technologies). Cells were harvested and fixed with BD CellFIX (BD Biosciences) for at least 30 minutes at 2–4°C and permeabilized in PBS 1% BSA 30% ethanol in the presence of Hoechst at a final concentration of 0.1 mg/ml. After 45 minutes of incubation at room temperature, cells were analyzed in a LSR-II flow cytometer (BD Bioscience) from the Centro de Citometría y Microscopía de Fluorescencia. Aggregates were excluded based on the width and the area of Hoechst's signal and the percentage of cells in the stages G_0_/G_1_ y S/G_2_/M was calculated.

### Apoptosis assays

Naive CD4^+^ T cells were cultured with monoclonal antibodies anti-CD3/anti-CD28 or in complete culture media alone, or supplemented with IL-7 [5 ng/ml], in the presence of DMSO or DMH1 at the indicated doses. The proportion of apoptotic cells was determined by staining with Annexin-V-FITC (BD Biosciences), according to the supplier’s instructions. Cells were analyzed on a FACSCalibur flow cytometer (Centro de Citometría y Microscopía de Fluorescencia) and gated according to forward scatter, side scatter, and their ability to exclude propidium iodide. Cell viability was calculated as percentage of Annexin-V-negative/propidium iodide-negative cells.

### Cytokine measurements

Culture supernatants of TCR-stimulated T cells were harvested at the indicated time points and levels of IL-2 were assayed by ELISA (BioLegend).

### Statistical analysis

The Student *t* test was used for statistical analysis. Values of p ≤ 0.05 (*), p ≤ 0.01 (**) and p ≤ 0.001 (***) were considered to be statistically significant.

## Results

### Induction of BMP signaling during TCR-triggered activation of naive CD4^+^ T cells

**Fig 1:**
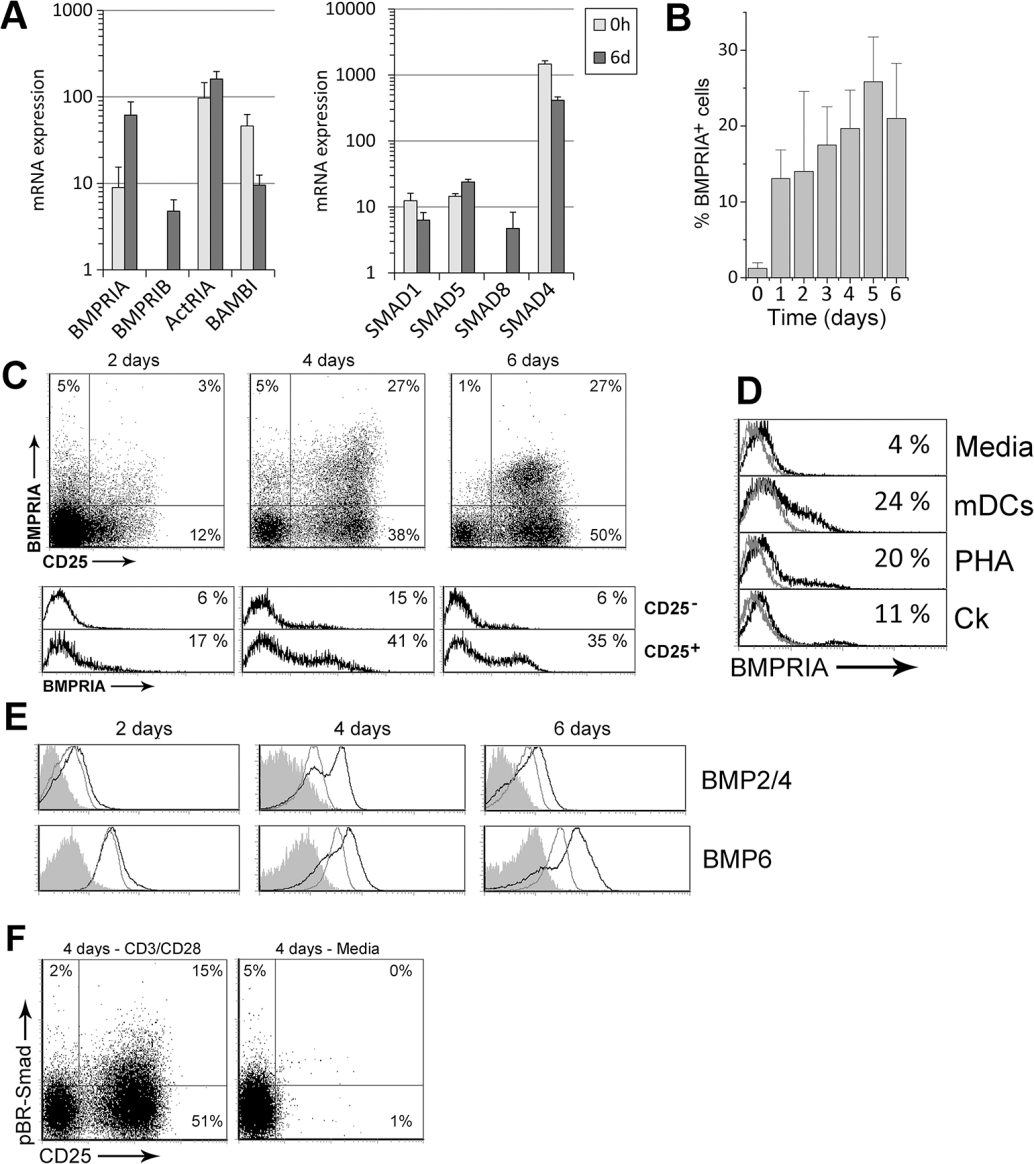
BMP signaling is activated by TCR stimulation in naive CD4^+^ T cells. Freshly isolated human peripheral blood naive CD4^**+**^ T cells were stimulated with anti-CD3/CD28 mAb. (A) Transcripts for several components of the canonical BMP signaling pathway were determined by real-time PCR ex vivo (0h) or after 6 days of stimulation (6d). *GNB2L1* was used as endogenous control. Means ± SD of at least three independent experiments run in duplicates are shown. Note the logarithmic scale on y-axis. (B) Percentage of BMPRIA^**+**^ cells detected by flow cytometry throughout the culture. Bars represent the mean ± SD of two to five independent experiments. (C) Expression of BMPRIA and CD25 in T cells (upper dot plots) and differential expression of BMPRIA in the CD25^**-**^ and CD25^**+**^ cell populations (lower histograms) during activation. A representative experiment out of four is shown. (D) Expression of BMPRIA in T cells cultured with different stimuli. Grey histograms represent isotype controls. Similar stainings were obtained in two to three independent experiments. mDCs: mature dendritic cells; PHA: Phytohaemagglutinin; Ck: cytokine cocktail (rhIL-2, rhIL-6, rhTNF-α) (E) Determination of BMP2/4 and BMP6 production by flow cytometry in T cells cultured in media alone (grey histograms) or in the presence of anti-CD3/CD28 mAb (black histograms). Grey filled histograms represent isotype control stainings. A representative experiment out of three is shown. (F) Expression of CD25 and phosphorylated BR-Smad (pBR-Smads) during activation. For comparison, T cells were kept in culture media alone. Results are representative of three independent experiments.

To investigate whether BMP signaling is activated in naive CD4^+^ T cells by TCR stimulation, we first analyzed the expression of several components of the canonical BMP pathway. We detected on freshly isolated naive CD4^+^ T cells the expression of the specific downstream effector molecules for the BMP signaling pathway, Smad-1, and -5, as well as the common Smad or Smad-4, that were also present after 6 days of activation with anti-CD3/CD28 mAbs (Fig 1A). In addition, transcription of the specific BMP receptors type I (BMPRIA, BMPRIB and ActRIA) and Smad-8 was induced in activated T cells while levels for the pseudoreceptor BAMBI were decreased compared with ex vivo (Fig 1A).

Among BMP receptors type I, BMPRIA is one of the most characterized. Its expression is found in several cell types including thymocytes [8, 11], peripheral CD4^+^ T cells and the Jurkat TAg cell line [13]. When the expression of BMPRIA was studied by flow cytometry in naive CD4^+^ T cells we found a rapid induction after only one day of culture with anti-CD3/CD28. BMPRIA expression was then increased throughout the culture reaching a maximum of 25% of positive cells at day 5 (Fig 1B). Furthermore, BMPRIA expression was mainly associated with that of the activation marker CD25, indicating that BMPRIA was preferentially expressed by those cells that responded to the stimulation (Fig 1C). We next investigated whether the induction of BMPRIA could be observed when other stimuli were used, finding that BMPRIA was induced in all cases. Besides treatment with PHA and co-culture with mature dendritic cells, BMPRIA surface expression was also slightly induced in naive CD4^+^ T cells by a TCR-independent stimulation such as exposure to pro-inflammatory cytokines (Fig 1D).

The production of BMP ligands in TCR-activated naive CD4^+^ T cells was also determined by intracellular flow cytometry staining at different time points. Both BMP2/4 and BMP6 production was induced by anti-CD3/CD28 stimulation when compared to unstimulated cells, following similar kinetics until 4 days of culture (Fig 1E), when approximately one third of the stimulated cells were positive for these ligands. However, while BMP2/4 expression tended to fade at day 6 of stimulation, BMP6 remained even higher compared to day 4 (Fig 1E).

As it could be expected, CD3/CD28 crosslinking efficiently induced the activation of the BR-Smads (Fig 1F). Phosphorylated BR-Smad (pBR-Smad) positive cells appeared preferentially in the CD25^+^ compartment after TCR stimulation and were absent in T cells cultured in media alone. Collectively, the results shown above suggest that TCR stimulation of human naive CD4^+^ T cells triggers the canonical BMP signaling in an autocrine fashion.

### BMPRIA expression pattern during CD4^+^ T cell activation

**Fig 2:**
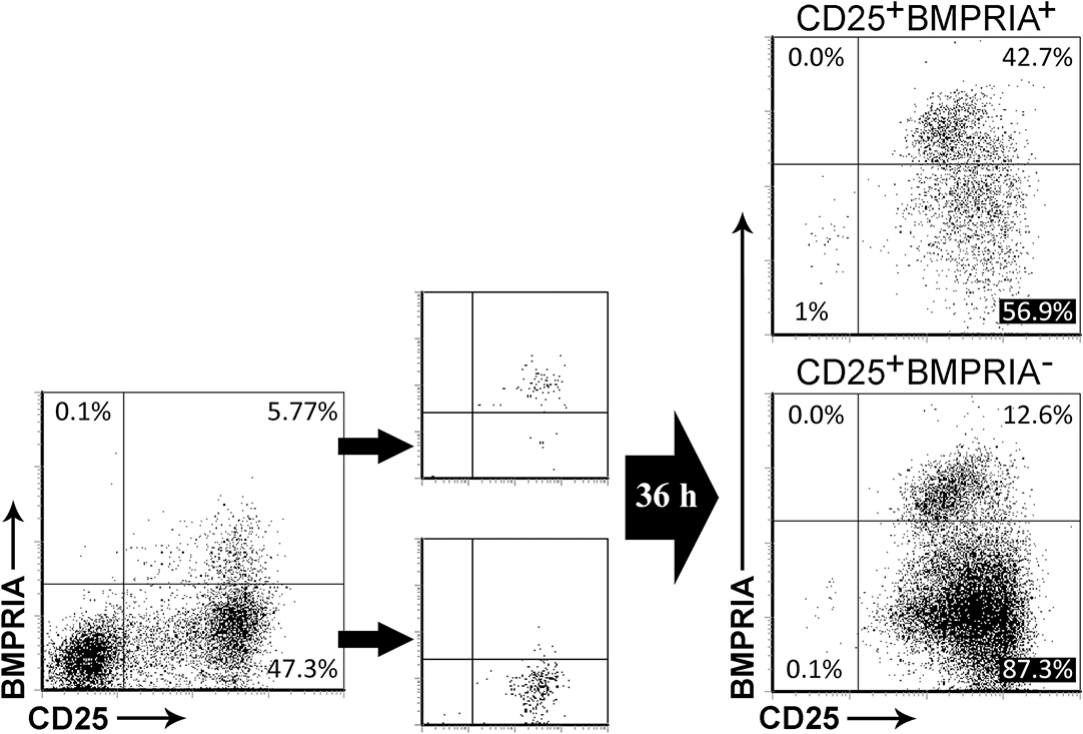
BMPRIA expression during CD4^+^ T cell activation. After 4 days of culture, TCR-activated T cells were stained for CD25 and BMPRIA and the CD25^**+**^BMPRIA^**-**^ and CD25^**+**^BMPRIA^**+**^ cell populations were isolated by cell sorting. The sorted populations were then re-stimulated with anti-CD3/CD28 mAb and the expression of CD25 and BMPRIA was evaluated by flow cytometry after 36 hours of culture. A representative experiment out of two is shown.

To test the possibility that a non-previously described minor subpopulation of naive CD4^+^ T cells expressing BMPRIA could be expanding during anti-CD3/CD28 stimulation, CD25^+^ T cells were sorted after 4 days of activation according to BMPRIA expression. As shown in Fig 2, after 36 hours of re-stimulation with anti-CD3/CD28 more than half of the sorted CD25^+^BMPRIA^+^ cells lost the expression of BMPRIA while maintaining that for CD25. On the other hand, more than 10% of BMPRIA^+^ cells appeared after re-stimulation of the CD25^+^BMPRIA^-^ cell subpopulation.

Endocytosis of BMP receptors once they bind their ligand is known to be a critical step for BMP signaling [27]. Moreover, BMP receptors undergo constitutive endocytosis in mammal cells [28]. We therefore analyzed the cytoplasmic expression of BMPRIA by flow cytometry and compared it with its surface expression. While very few naive CD4^+^ T cells exhibited BMPRIA in their surface *ex vivo*, the majority of this population of T cells was positive for BMPRIA when intracellular staining was performed (Figure A in S1 Fig). These results were confirmed by immunofluorescence (Figure B in S1 Fig) and suggest that the percentage of cells which potentially could respond to BMP ligands seems to exceed what would be expected by only analyzing the surface expression of BMPRIA.

### Autocrine canonical BMP signaling regulates TCR-induced proliferation in naive CD4^+^ T cells

**Fig 3:**
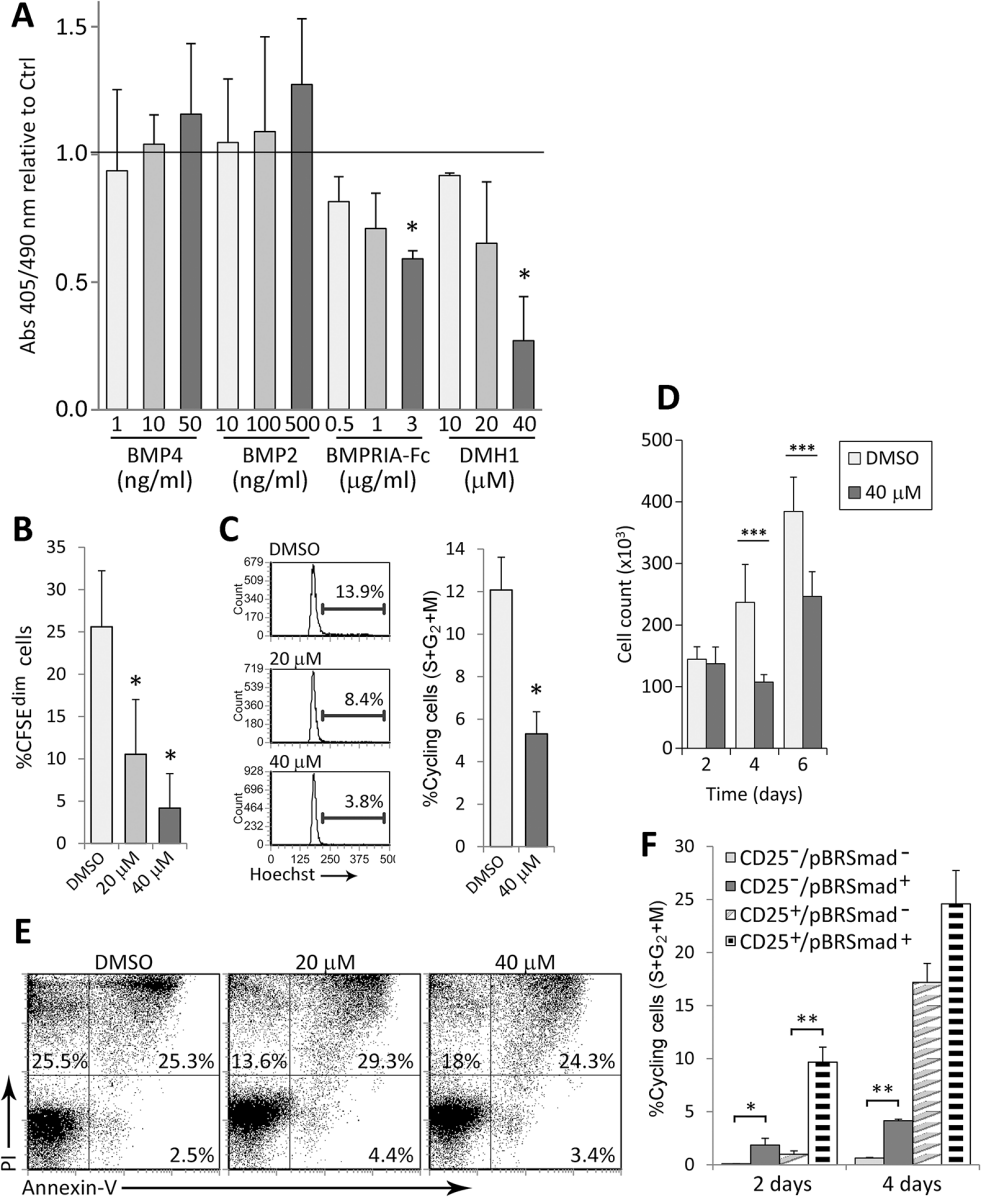
Canonical BMP pathway inhibition impairs T cell proliferation. (A) BrdU incorporation after 4 days of TCR stimulation with the indicated treatments. Results are represented as absorbance relative to control (horizontal bar). Means ± SD of two independent experiments run in duplicates are shown (* p≤0.05; by *t* test). Control refers to no treatment for BMP2 and BMP4, unspecific immunoglobulins for BMPRIA-Fc and DMSO for DMH1. (B-D) After 4 days of TCR stimulation in the presence of DMSO or DMH1, the proliferation rate, measured by CFSE loss (B), Hoechst staining (C) and number of cells recovered (D), and the percentage of apoptotic/necrotic cells (E) were analyzed by flow cytometry. Bars represent means ± SD of five independent experiments (* p≤0.05; by *t* test). Cell counts were performed in duplicates and the means ± SD of at least 4 independent experiments are shown. Histograms in (C) and dot plots in (E) correspond to one representative experiment. (F) Cells were harvested at the indicated time points and stained for CD25, phosphorylated Smad-1/5/8 (pBR-Smad) and Hoechst. Cell populations were defined according to the expression of CD25 and pBR-Smad and the percentage of Hoechst positive cells was determined for each subset. Means ± SD of three independent experiments are shown (* p≤0.05; ** p≤0.01; by *t* test).

As a first approach, we analyzed whether modifications of BMP signaling could affect the proliferation of T cells. BMP signaling was activated by addition of exogenous BMP2 and BMP4, the only two members of the BMP2/4 subgroup which has shown the highest affinity for BMPRIA [2]. In parallel, BMP signaling was inhibited by two means: blocking soluble BMPs by addition of the recombinant human BMPR-IA/ALK-3 Fc chimera, and inhibiting BR-Smad phosphorylation by BMP receptors type I with the inhibitor DMH1 [26]. As shown in Fig 3A, the addition of BMP2 and BMP4 slightly increased BrdU incorporation at day 4 of culture, although no statistical significance was reached. On the contrary, and supporting an autocrine role for BMP signaling pathway, both BMPRIA-Fc and DMH1 negatively affected the proliferative response of T cells, being this effect greater with the BMP inhibitor DMH1 (Fig 3A). The impairment of proliferation induced by BMP signaling blockade was confirmed by CFSE loss, showing that the presence of the BMP inhibitor DMH1 reduced the proliferative response induced by anti-CD3/CD28 stimulation by nearly an 80% with the highest dose (Fig 3B). This effect was dose-dependent and mainly caused by G_0_/G_1_ arrest, since the percentage of cycling cells was reduced by more than half in DMH1-treated cells (Fig 3C). Accordingly, the effects on cell cycle progression induced by DMH1 correlated well with the number of cells recovered after stimulation, being these numbers markedly lower in DMH1-treated cultures compared to control cultures (Fig 3D). It must be noted that the inhibition of proliferation induced by DMH1 treatment was not caused by exacerbated apoptosis, neither by toxic effects, since no remarkable differences were observed when cell viability was analyzed (Fig 3E).

To study more deeply the relationship between BMP signaling and T cell proliferation, we performed the detection of intracellular pBR-Smads combined with the surface expression of CD25 and DNA staining with Hoechst. Supporting the positive role of the canonical BMP signaling in TCR-induced T cell proliferation, the expression of pBR-Smads correlated with cell cycle progression (Fig 3F). Specifically, a higher percentage of cycling cells was found within the pBR-Smad^+^ compartment in both the CD25^-^ and CD25^+^ cell populations at day 2. In contrast, after 4 days of culture, when IL-2 levels are elevated, only the CD25^-^ cell population showed statistically significant correlation between pBR-Smads expression and Hoechst staining (Fig 3F).

### IL-2 production is impaired by inhibition of autocrine BMP signaling during T cell activation

**Fig 4:**
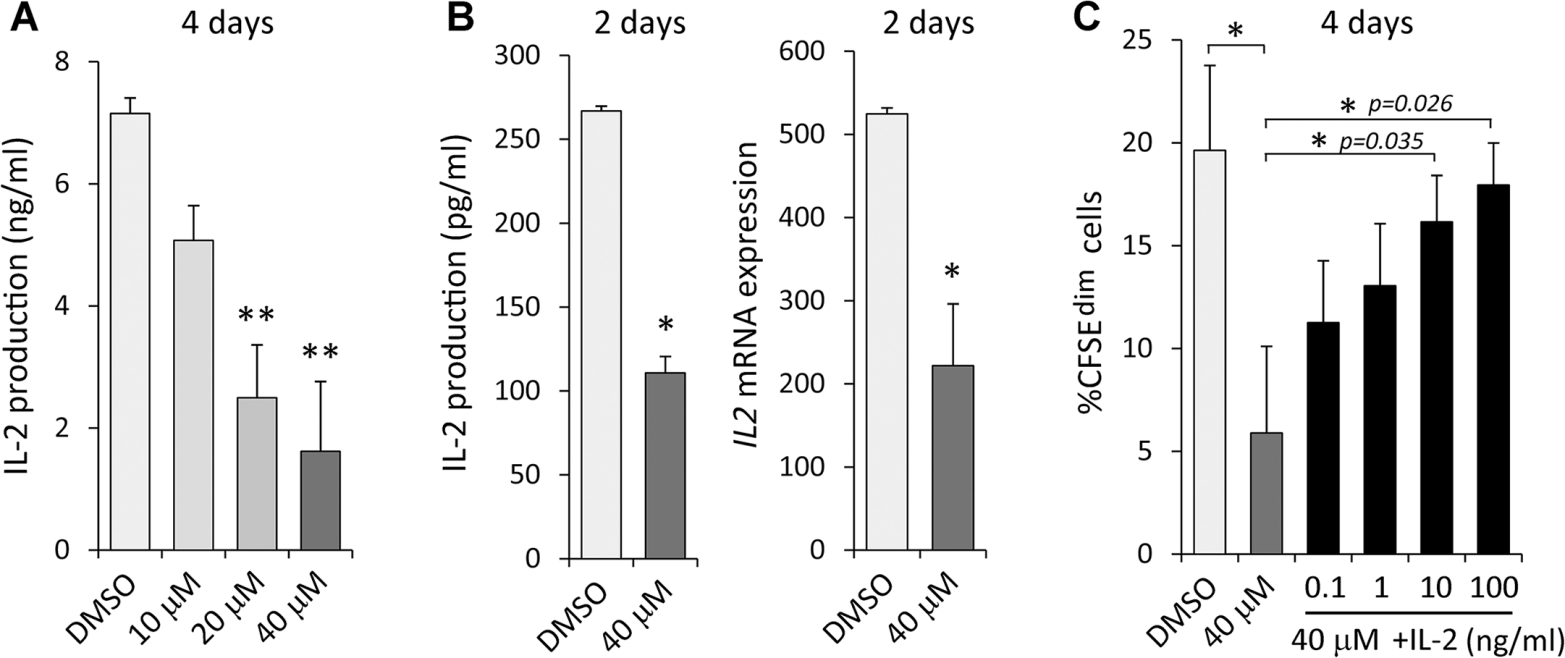
DMH1 effects on IL-2 expression. TCR-induced IL-2 production by T cells in the presence of DMSO or DMH1 at day 4 (A) and 2 (B, left graph). (B, right graph) mRNA expression for *IL2* after 2 days of activation. *GNB2L1* was used as endogenous control. Means ± SD of three to five independent experiments performed in duplicates are shown (* p≤0.05; ** p≤0.01; by *t* test). (C) Proliferation rate measured by CFSE loss in T cells after 4 days of TCR stimulation with DMSO, DMH1 alone or DMH1 supplemented with the indicated doses of rhIL-2. Bars represent the means ± SD of three independent experiments (* p≤0.05; by *t* test).

Secretion of IL-2 by T cells during activation is crucial for their own proliferation. We therefore determined the production of IL-2 in these cultures finding that DMH1 treatment had a negative impact on IL-2 levels after 4 days of culture. Specifically, canonical BMP signaling blockade affected IL-2 production in a dose-dependent manner reaching a maximum of 80% of inhibition when the highest dose of DMH1 was used (Fig 4A). In addition, *IL2* transcription as well as IL-2 protein secretion were impaired by DMH1 treatment as soon as 2 days after TCR stimulation (Fig 4B). On the contrary, no effects in the expression of CD25 were observed at this time point (data not shown). To better understand the role of IL-2 in the inhibition induced by DMH1, rhIL-2 was added at different doses together with DMH1 and the proliferative response was measured by CFSE loss. As expected, increasing concentrations of rhIL-2 partially counteracted the inhibiting effects of DMH1, being the highest dose of rhIL-2 able to fully restore the proliferative response of T cells (Fig 4C).

### BMP signaling is autocrinally triggered by IL-7 in naive CD4^+^ T cells

**Fig 5:**
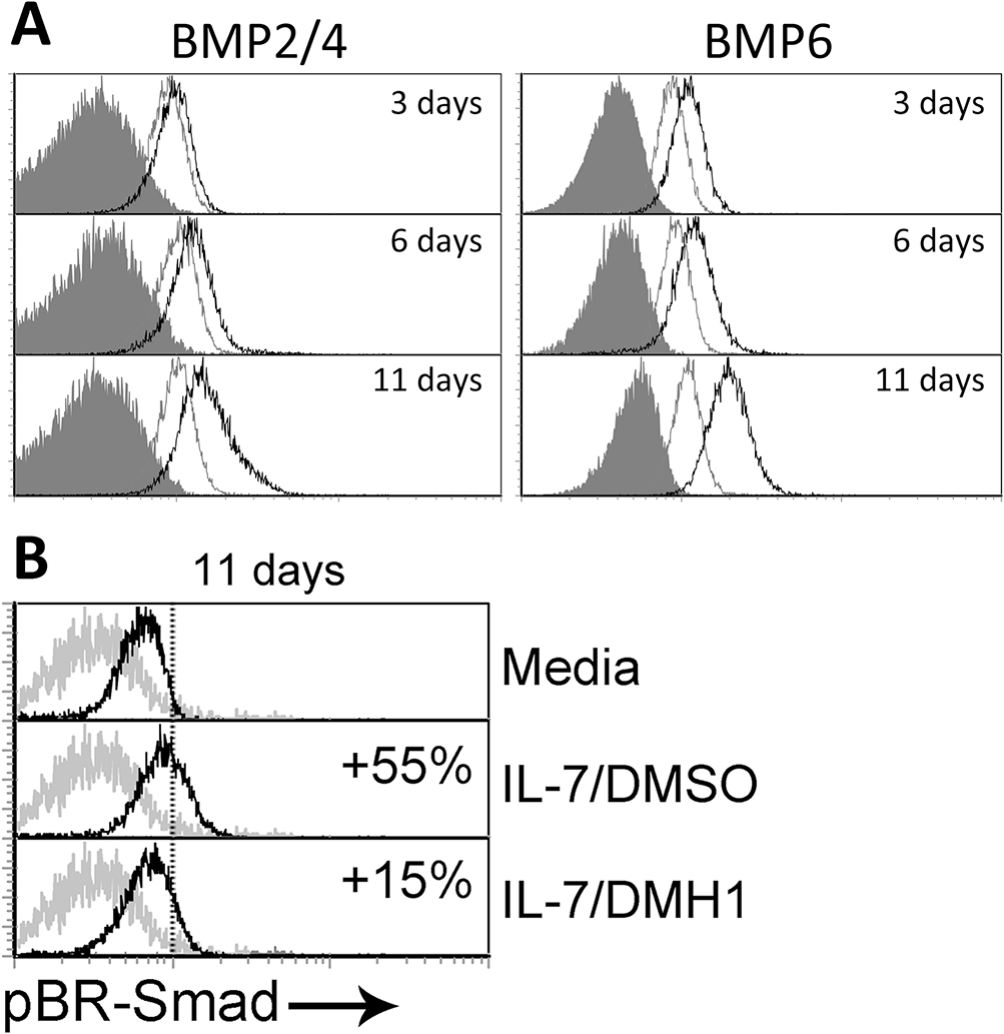
Canonical BMP pathway and IL-7 signaling. (A) Expression of BMP2/4 and BMP6 was determined by flow cytometry at the indicated time points in T cells cultured in media alone (grey histograms) or in the presence of IL-7 (5 ng/ml) (black histograms). Grey filled histograms represent isotype control stainings. A representative experiment out of four is shown. (B) Differential expression of phosphorylated Smad-1/5/8 (pBR-Smad) analyzed by flow cytometry in naive CD4^**+**^ T cells after 11 days of culture in media alone or supplemented with IL-7 and DMSO or IL-7 and DMH1 (40 μM). Percentages represent the increment relative to cultures in media alone. One representative of three independent experiments is shown.

Our results pointed out a tight relationship between autocrine BMP signaling and activation-induced T cell proliferation. Hence, we next wondered whether the BMP signaling pathway could be acting in a different process also associated with proliferation. In this regard, IL-7 is a well known key factor regulating the homeostasis of naive and memory T cells and capable of inducing the so-called homeostatic proliferation of both subsets [20]. When naive T cells were cultured in the presence of IL-7, production of BMP ligands BMP2/4 and BMP6 was progressively induced throughout the culture (Fig 5A). Expression of these ligands showed a homogeneous pattern suggesting that the majority of the cells were responding to IL-7 stimulation in terms of BMP production. Accordingly, levels of pBR-Smads were increased by 55% compared to cells cultured in media alone at day 11, while addition of the DMH1 inhibitor mostly inhibited BR-Smad phophorylation (Fig 5B).

### Canonical BMP signaling is necessary for IL-7-induced homeostatic proliferation

**Fig 6:**
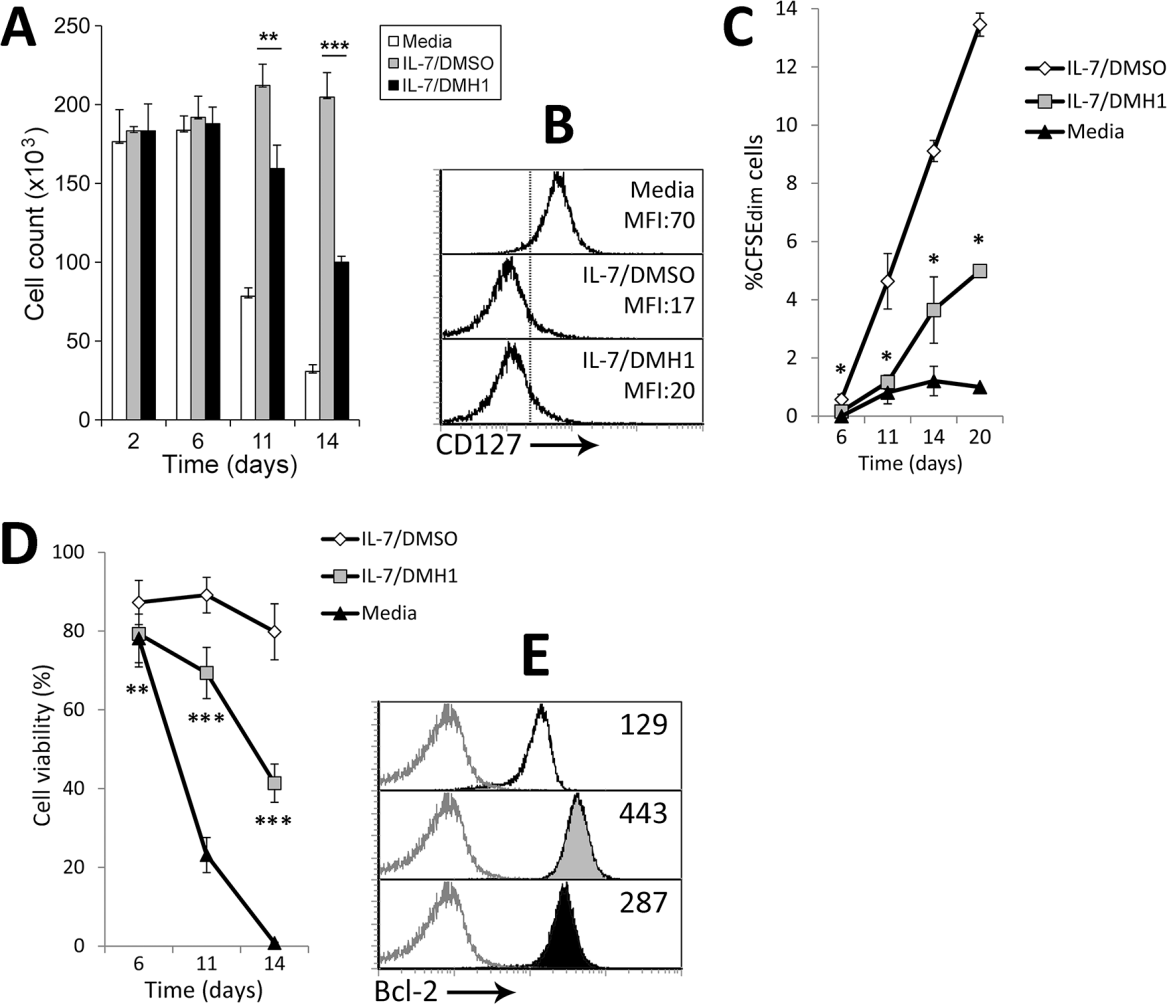
Effects of BMP pathway blockade on IL-7-induced T cell homeostasis. (A) Naive CD4^**+**^ T cells cultured in media alone or supplemented with IL-7 and DMSO or IL-7 and DMH1 (40 μM) were harvested and counted at the indicated time points. Cell counts were performed in duplicates. Results represent the mean ± SD of four to twelve samples pooled from at least two independent experiments (** p≤0.01; *** p≤0.005; by *t* test. IL-7/DMSO vs IL-7/DMH1). (B) Differential expression of CD127 analyzed by flow cytometry after 36 hours of culture under the indicated conditions. Similar stainings were obtained in two independent experiments. (C) Proliferation rate measured by CFSE loss along 20 days in T cells cultured in media alone or supplemented with IL-7 and DMSO or IL-7 and DMH1 (40 μM). Means ± SD of four independent experiments are shown (* p≤0.05; by *t* test. IL-7/DMSO vs IL-7/DMH1). (D) Cell viability calculated as percentage of PI^**-**^/Annexin-V^**-**^ cells throughout the culture. Means ± SD of four independent experiments are shown (** p≤0.01; *** p≤0.005; by *t* test. IL-7/DMSO vs IL-7/DMH1). (E) Bcl-2 levels determined by flow cytometry after 6 days of culture. White filled histograms represent media alone; grey-filled IL-7/DMSO; black-filled IL-7/DMH1. The mean fluorescence intensity is indicated in each histogram. Similar stainings were obtained in two independent experiments.

In order to analyze the implication of the BMP pathway in IL-7-induced T cell homeostasis, we first studied the progression of IL-7 cultures in terms of cell numbers. As shown in Fig 6A, while recovery of naive CD4^+^ T cells cultured in media alone tended to rapidly decrease, cell numbers in cultures supplemented with IL-7 were even slightly increased. Nevertheless, in the presence of the BMP inhibitor DMH1, stimulation with IL-7 failed to maintain cell numbers throughout the culture, revealing a role for the BMP pathway in IL-7-induced homeostasis. Remarkably, the addition of DMH1 to these cultures did not affect the downregulation of the high affinity IL-7 receptor CD127 that normally occurs when IL-7 signaling is triggered, suggesting that activation of IL-7 signaling was not being impaired (Fig 6B).

The role of the autocrine canonical BMP signaling in the proliferation of naive CD4^+^ T cell induced by IL-7 was studied in long term cultures. In line with the inhibitory effect of DMH1 treatment on TCR-induced proliferation, the presence of the BMP inhibitor also caused a marked reduction in the proliferative response induced by IL-7 along 20 days of culture (Fig 6C). Proliferation of DMH1-treated cells was minimal until day 14 of stimulation, when treated cells began to proliferate always in a lesser extent compared to that observed in DMSO-treated cells (Fig 6C).

### Canonical BMP signaling inhibition impairs the survival of naive CD4^+^ T cells induced by IL-7

Because IL-7 is known to be an essential cytokine for the survival of T cells, we studied whether DMH1 treatment could impact the viability of these cells cultured with IL-7. Blockade of canonical BMP signaling during IL-7 stimulation severely affected the survival of T cells along the culture period decreasing the percentage of viable cells from 80% in control DMSO-treated cultures to 40% in DMH1-treated cultures after 14 days of stimulation (Fig 6D). Mechanistically, we found that levels of the anti-apoptotic protein Bcl-2, commonly induced by IL-7, were decreased in the presence of DMH1 from the beginning of these cultures (Fig 6E), indicating that autocrine BMP signaling would participate in the regulation of IL-7-induced T cell survival through modulation of Bcl-2.

### BMP signaling inhibition affects the modulation of homing receptors induced by IL-7

**Fig 7:**
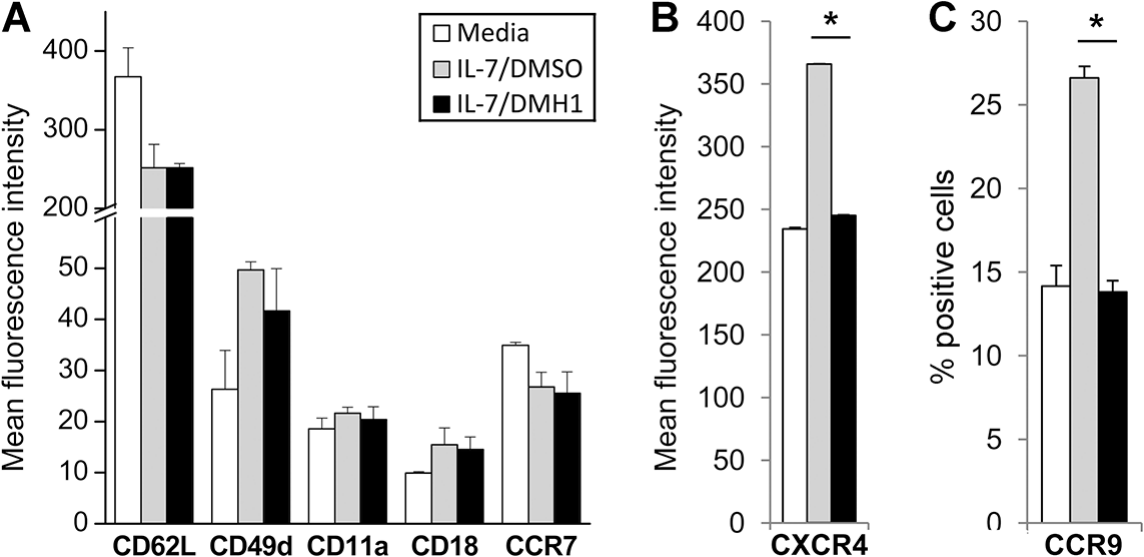
DMH1 effects on IL-7-induced homing receptor modulation. T cells were cultured in media alone or supplemented with IL-7 and DMSO or IL-7 and DMH1 (40 μM) and the expression of several homing receptors (A), CXCR4 (B) and CCR9 (C) was analyzed by flow cytometry after 36 hours of culture. Bars represent the mean ± SD of two independent experiments (* p≤0.05; by *t* test. IL-7/DMSO vs IL-7/DMH1).

Given that IL-7 has been described to modulate the expression of several homing receptors in T cells [29], we analyzed whether autocrine BMP signaling could be mediating in this process. DMH1 treatment showed no effects in the regulation of the lymph node homing receptors L-selectin/CD62L and the integrins α4/CD49d, αL/CD11a and β2/CD18 (Fig 7A). Similar results were obtained for the chemokine receptor CCR7, shown to be important for lymphocyte migration towards lymph nodes (Fig 7A). According to previous results, IL-7 induced the upregulation of the chemokine receptor CXCR4. Nevertheless, CXCR4 induction was completely abrogated in the presence of DMH1 (Fig 7B). In the same line, IL-7 induction of the gut homing receptor CCR9 was also abolished by canonical BMP signaling inhibition (Fig 7B), showing a very specific impact of this inhibitor on the regulation of homing receptors provoked by IL-7.

## Discussion

The BMPs have emerged during the last decades as multifunctional proteins which regulate essential processes such as differentiation, proliferation and survival in a wide range of cell types. In this study, we describe that BMP signaling mediates the regulation of the proliferation of human naive CD4^+^ T cells induced during two crucial events for the biology of these cells: activation and homeostasis.

Regarding T cell activation, we have found that T cells increase the expression of BMP signaling components in response to TCR stimulation. Surface expression of BMPRIA was quickly induced by CD3/CD28 crosslinking and was mainly associated to those cells that responded to the stimulation. Furthermore, we show for the first time that human CD4^+^ T cells are capable of producing the ligands BMP2, BMP4 and BMP6 in response to TCR stimulation. Notably, expression of activated BR-Smads was induced upon TCR stimulation, which confirmed that canonical BMP signaling is autocrinally triggered during the activation of T cells. Co-culture with mature dendritic cells and TCR-independent stimulation also induced surface expression of BMPRIA. These results suggest that upregulation of BMP signaling could be an inherent mechanism induced by T cell activation independently of the stimulus. Supporting this data, a previous work showed low expression levels for several BMP receptors in human peripheral blood CD4^+^ T cells, being this expression greater in the CD45RA^-^ memory T cell compartment [13]. Interestingly, intracellular staining revealed that the moderate percentage of BMPRIA^+^ resting CD4^+^ T cells could be explained by preferential localization of this protein in intra-vesicular compartments rather than at the cell surface. This phenomenon has also been described in human peripheral blood NK cells [30] and is in line with the fact that BMP receptors undergo constitutive endocytosis even in the absence of ligand [28].

Evidences of a pro-activation role of BMPs in T cells can be found in the literature. For instance, mouse CD4^+^ T cells primed in the presence of BMP2 or BMP4 showed higher proliferation when re-stimulated with antigen presenting cells [12]. Also in the mouse model, different members of the TGF-β superfamily, including BMPs, are able to increase the production of IFN-γ by CD8^+^ T cells that takes place during antigen specific responses [14]. Our results show that the proliferative response of naive CD4^+^ T cells is regulated at least in part by autocrine canonical BMP signaling. Both ligand neutralization and blockade of BMP receptor kinase activity had a dose-dependent negative effect in the proliferative ratio, mainly caused by G_0_/G_1_ arrest, and not by an increment in apoptosis. Furthermore, active BMP signaling was strongly associated with cell cycle progression as early as two days after TCR activation. This association was partially lost in the CD25^+^ cell population after 4 days of stimulation, when the IL-2/IL-2R positive feedback is the principal mechanism driving T cell proliferation [31, 32]. This suggests that activation of BMP signaling would be advantageous during the early steps of activation while would be overcome by IL-2 signaling at later steps. Supporting this idea, we show that BMP signaling blockade results in a strong inhibition of IL-2 expression at both mRNA and protein levels. Moreover, proliferation of DMH1-treated T cells is completely recovered when rhIL-2 is added to these cultures. Yoshioka et al [33] previously reported in mice that treatment with the inhibitor of BMP signaling dorsomorphin also affects the proliferation of T cells induced by TCR stimulation through impairment of IL-2 production. The authors and others have pointed out Runx1, a target gene for BMP signaling, as the transcription factor regulating IL-2 expression in these cells [34], although contradictory results have been published [35]. Also in support for the regulation of IL-2 expression by BMP signaling, it has been shown that BMP2 and BMP4 can increase the frequency and activity of Treg, a Th subset strongly dependent on IL-2 signaling [12]. Taken together, these evidences indicate a role of the canonical BMP signaling in the regulation of T cell proliferation in response to anti-CD3/CD28 stimulation via modulation of IL-2 expression.

The present study denote that autocrine BMP signaling is likewise involved in the proliferation of human CD4^+^ naive T cells mediated by IL-7, a crucial factor for the homeostasis of these cells [20]. IL-7 stimulation induced BMP2, 4 and 6 production and the consequent activation of BR-Smads on T cells, which was abolished when DMH1 was present. Inhibition of BMP signaling negatively affected the IL-7-regulated homeostasis of T cells by two means: reducing cell survival, one of the hallmarks of IL-7 signaling on T cells, and impairing the so-called homeostatic proliferation. A direct role of BMPs regulating cell proliferation has been extensively described in several cell types including thymocytes [8, 9], B cells [36] and mature T cells [12, 13, 33]. Among the mechanisms mediating in this process, several authors have pointed out the cell cycle inhibitor p27kip1 as a target for BMP signaling [33, 37–41]. Furthermore, p27kip1 has been shown to play a crucial role in the homeostatic proliferation induced by IL-7 on T cells and T-cell acute lymphoblastic leukemia cells [42, 43]. These evidences may serve as a rational explanation for the lack of proliferation provoked by BMP signaling blockade, although further research will be necessary to prove them right.

On the other hand, autocrine BMP signaling is also involved in the regulation of IL-7-induced cell survival, since DMH1 treatment severely affects the cell viability induced by this cytokine. This effect was probably caused by a failure to upregulate Bcl-2, an anti-apoptotic protein normally induced by IL-7 [44] that is frequently targeted by BMP signaling [45–47]. In addition, either delayed induction or enhanced degradation of Bcl-2 would explain the observed reduction in Bcl-2 protein levels when BMP signaling was inhibited. Of note, addition of BMP4 slightly increased the survival provoked by IL-7 (data not shown), showing opposite effects to those observed when BMP signaling was blocked.

In addition, we have found that the upregulation of the chemokine receptors CXCR4 and CCR9 induced by IL-7 is specifically impaired in the presence of DMH1. Among the range of responses that characterized IL-7 stimulation on T cells, it has been described that this factor could mediate specific T cell migration through the modulation of a number of homing receptors [29]. Our results would suggest the involvement of the BMP pathway in the acquisition of certain migratory capacities in response to IL-7 [48, 49], although further experiments will be required to address this possibility.

The results shown in this study reveal an important role of the canonical BMP signaling in human naive CD4^+^ T cell biology that could be valuable for clinical application. On one hand, the implication of BMP signaling in the earliest phases of T cell activation could provide a therapeutic target for lymphoproliferative and autoimmune syndromes, such us graft-versus-host disease and rheumatoid arthritis, in which control of T cell activation is essential. On the other hand, given the direct role that IL-7 signaling plays in augmenting reactivity to self-antigens during lymphopenia because of excessive levels of IL-7 [50], manipulation of BMP signaling might be considered for prevention of autoimmune diseases in lymphopenic individuals.

## Supporting Information

S1 Fig Surface and intracellular immunostaining for BMPRIA in *ex vivo* and stimulated naive CD4^+^ T cells. (Figure A) Determination by flow cytometry of surface (upper dot plots) and intracellular (lower dot plots) BMPRIA expression in CFSE-stained T cells before and after stimulation via TCR. Percentage of CFSE^dim^BMPRIA^+^ cells is shown. Results are representative of three independent experiments. (Figure B) Isolated CD4^+^ T cells were attached to poly-L-lysine coated glass slides and stained for surface CD4 and surface (upper panels) and intracellular (lower panels) BMPRIA and imaged by confocal microscopy. Similar stainings were obtained in three independent experiments.
(TIF)
