# Supplementary figures and images for: The BMP Pathway Participates in Human Naive CD4+ T Cell Activation and Homeostasis
Source: PLoS One. 2015 Jun 25;10(6):e0131453. doi: 10.1371/journal.pone.0131453 (PMC4481406; doi:10.1371/journal.pone.0131453)

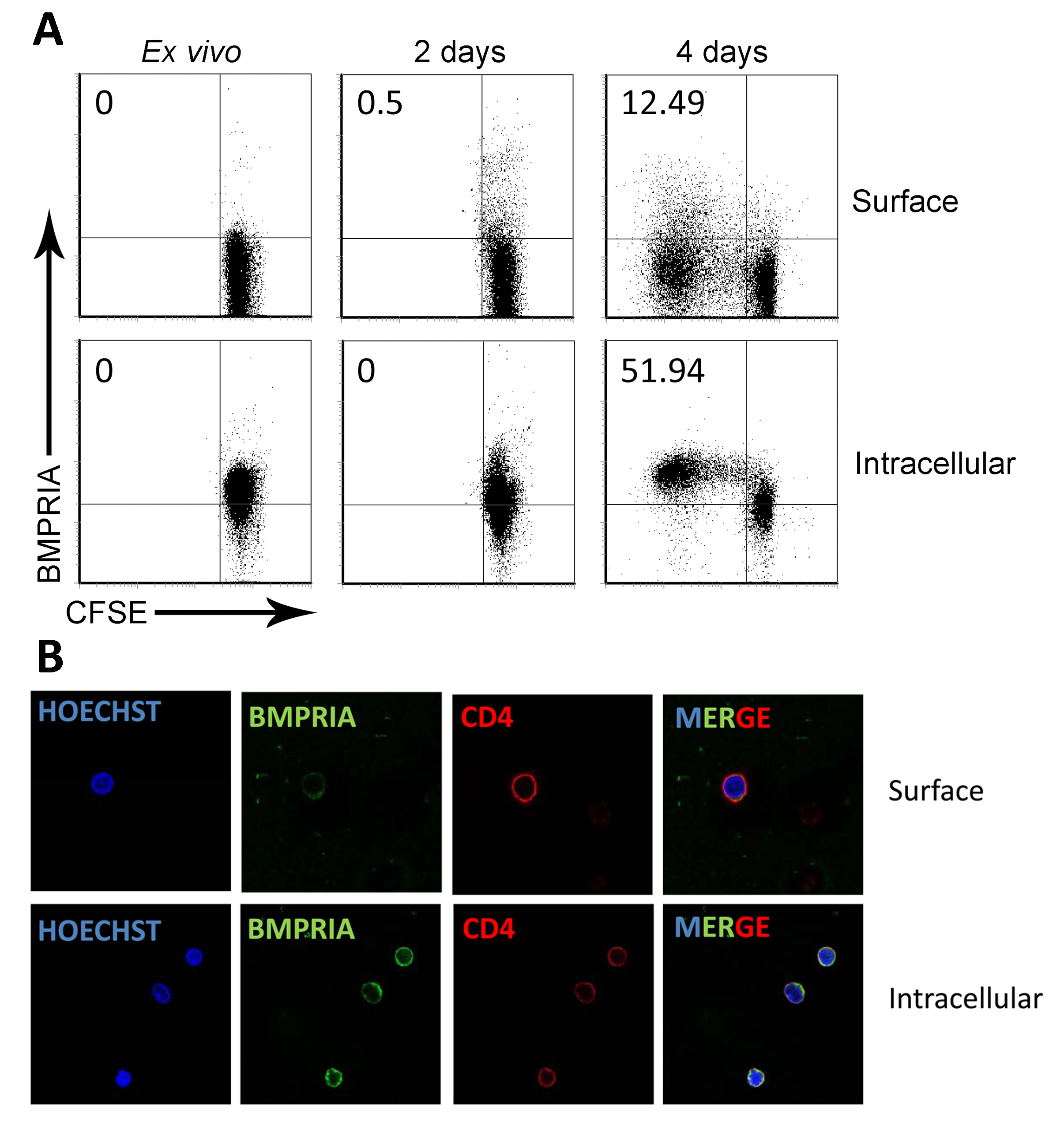

Supplement: S1 Fig — (Figure A) Determination by flow cytometry of surface (upper dot plots) and intracellular (lower dot plots) BMPRIA expression in CFSE-stained T cells before and after stimulation via TCR. Percentage of CFSEdimBMPRIA+ cells is shown. Results are representative of three independent experiments. (Figure B) Isolated CD4+ T cells were attached to poly-L-lysine coated glass slides and stained for surface CD4 and surface (upper panels) and intracellular (lower panels) BMPRIA and imaged by confocal microscopy. Similar stainings were obtained in three independent experiments. (TIF) [file pone.0131453.s001.tif]
